# Simplifying Consent for HIV Testing Is Associated with an Increase in HIV Testing and Case Detection in Highest Risk Groups, San Francisco January 2003–June 2007

**DOI:** 10.1371/journal.pone.0002591

**Published:** 2008-07-02

**Authors:** Nicola M. Zetola, Carlos G. Grijalva, Sarah Gertler, C. Bradley Hare, Beth Kaplan, Teri Dowling, Grant Colfax, Mitchell H. Katz, Jeffrey D. Klausner

**Affiliations:** 1 Division of Infectious Diseases, University of California San Francisco, San Francisco, California, United States of America; 2 Department of Preventive Medicine, Vanderbilt University School of Medicine, Nashville, Tennessee, United States of America; 3 School of Medicine, University of California San Francisco, California, United States of America; 4 Positive Health Program, University of California San Francisco, San Francisco, California, United States of America; 5 Department of Emergency Medicine, University of California San Francisco, San Francisco, California, United States of America; 6 San Francisco Department of Public Health, San Francisco, California, United States of America; Instituto de Medicina Tropical Alexander Von Humboldt, Peru

## Abstract

**Background:**

Populations at highest risk for HIV infection face multiple barriers to HIV testing. To facilitate HIV testing procedures, the San Francisco General Hospital Medical Center eliminated required written patient consent for HIV testing in its medical settings in May 2006. To describe the change in HIV testing rates in different hospital settings and populations after the change in HIV testing policy in the SFDH medical center, we performed an observational study using interrupted time series analysis.

**Methods:**

Data from all patients aged 18 years and older seen from January 2003 through June 2007 at the San Francisco Department of Public Health (SFDPH) medical care system were included in the analysis. The monthly HIV testing rate per 1000 hadpatient-visits was calculated for the overall population and stratified by hospital setting, age, sex, race/ethnicity, homelessness status, insurance status and primary language.

**Results:**

By June 2007, the average monthly rate of HIV tests per 1000 patient-visits increased 4.38 (CI, 2.17–6.60, p<0.001) over the number predicted if the policy change had not occurred (representing a 44% increase). The monthly average number of new positive HIV tests increased from 8.9 (CI, 6.3–11.5) to 14.9 (CI, 10.6–19.2, p<0.001), representing a 67% increase. Although increases in HIV testing were seen in all populations, populations at highest risk for HIV infection, particularly men, the homeless, and the uninsured experienced the highest increases in monthly HIV testing rates after the policy change.

**Conclusions:**

The elimination of the requirement for written consent in May 2006 was associated with a significant and sustained increase in HIV testing rates and HIV case detection in the SFDPH medical center. Populations facing the higher barriers to HIV testing had the highest increases in HIV testing rates and case detection in response to the policy change.

## Introduction

### Background

Populations at highest risk for HIV infection in the United States still exhibit the greatest gap between intention to test and actual testing [1]. Integration of HIV screening into routine medical care and the elimination of structural barriers to testing have the potential to increase overall HIV testing and case detection, particularly among populations at highest risk for HIV infection [2], [3].

In May 2006, the San Francisco Department of Public Health (SFDPH) medical care system eliminated the requirement for separate written consent for HIV testing within medical settings [4]. We previously reported that the policy change was associated with a significant increase in HIV testing rates and HIV case detection [4]. However, it was still unclear how that policy change affected different subpopulations and whether that increase would be sustained beyond the first few months after the change in policy went into effect.

## Methods

### Policy change

Before May 15, 2006 clinicians in the SFDPH medical care system were required to complete a separate HIV test laboratory requisition form and obtain a patient's signature on an informed consent document to order an HIV test. The laboratory rejected samples with incomplete documentation. Beginning on May 16 2006, patient consent forms were removed from medical settings and HIV antibody testing was added to the routine laboratory requisition form. Consistent with California State law, clinicians were required to document in the medical record that informed consent was obtained, but a patient's signature was no longer required [4].

### Data Source

All data were obtained from The Health Records Electronic Data Set (THREDS) from the University of California, San Francisco (UCSF) Clinical and Translational Science Institute (CTSI) Clinical Research Center. The present study included all patients aged 18 years or older, seen at the SFDPH medical center from January 2003 through June 2007.

### Definitions

An HIV test was defined as any HIV antibody test processed by the SFDPH medical center Clinical Laboratory during the study period. Confirmatory assays (HIV western blot and/or immunofluorescence assays), inadequate specimens (where no further HIV tests were recorded within 7 days), and cancelled HIV tests were excluded from the analysis. When a rejected test was followed by an HIV test with a valid result (e.g., positive, negative or indeterminate) within the following 30 days after testing, only the HIV test with a valid result was included in the analysis. Rejected tests that were not followed by a valid HIV test within the following four weeks were considered “true rejections” and included in the analysis. Information specifying lack of consent documentation as the reason for test rejection was collected when available. New HIV positive tests were defined as an HIV positive antibody test result confirmed by a positive HIV-1 western blot or immunofluorescence assay, and without a prior positive HIV test in our database. We included no more than one test per patient per month, giving priority to HIV tests with valid results. HIV testing rates were calculated as HIV tests ordered per 1000 patient-visits per month. Unlike our previous report [4], in which all patient-visits to the entire health care system were included in the denominator, this report only includes patient-visits to health care settings in which HIV screening was routinely performed. Health care setting where HIV screening is routinely performed included the emergency department, urgent care clinic, inpatient services, primary care clinics, specialty clinics and affiliated community clinics, but excluded affiliated long term facilities and nursing homes. Although this new approach might lead to higher monthly HIV testing rates than the ones reported previously by our group [1], we believe these rates reflect more accurately the HIV screening practices at our institution.

To assess whether changes in HIV testing within the SFDPH medical care system could be explained by changes in laboratory practices not related to the policy change, the trend of HIV testing rates was compared to the use of other blood tests that were not expected to be affected by the policy change (i.e. serum creatinine, sodium and hematocrit). Similarly, to test whether changes in HIV testing were specific to settings where the policy change was implemented, we compared the monthly HIV testing rates at our institution against those of another large university-based medical center in San Francisco in which the policy change did not occur. Although information on the number of monthly patient-visits to that medical center was not available, we used the number of laboratory requests as a surrogate. HIV testing rates for that facility were calculated as HIV tests ordered per 10,000 samples tested at laboratory per month.

### Study design and statistical analysis

We hypothesized that populations with higher rates of HIV test rejection due to inappropriate consent documentation prior to the policy change and populations with higher rates of HIV positive test results would have higher increases in monthly HIV testing rates after the elimination of the required written consent. Therefore, we identified factors associated with increased likelihood of having a rejected HIV test or a new HIV positive test result by logistic regression. All variables included in the logistic regression analysis were determined a priori based on estimation of their significance as epidemiological factors during the preliminary crude analysis (significant at *p*≤0.05) and biological plausibility. The model included the following variables: sex, age category, insurance status, homelessness status, race/ethnicity, primary language, and testing venue.

To determine the effect of the policy change on the HIV testing rates per 1000 patient-visits in different subpopulations, data were analyzed through interrupted time-series analyses. The study period (53 calendar months) was divided into “before” (40 months) and “after” policy change (13 months) segments. The month of policy change (May 2006) was considered a transition month and excluded from the analysis. Segmented regression analyses were used to measure the effect of the policy change [5], [6]. The regression models included terms for the policy change and secular trends for the periods before and after the policy change. Because error terms of consecutive observations were correlated, all analyses accounted for first order autocorrelation through auto regressive integrated moving average (ARIMA) and auto distributive lag (ADL) models. Residual analyses of the final models showed no significant deviations from model assumptions [5], [6].

A two-sided p<0.05 was considered statistically significant and analyses were performed using STATA version 8.2 (StataCorp Inc, College Station, Texas). The University of California San Francisco Committee on Human Research approved this study and waived patient consent requirements.

## Results

A total of 20,710 HIV tests were performed at the SFDPH medical center from January 2003 through June 2007 (Table 1). Before the policy change was implemented, 814 (68%) out of the 1204 rejected HIV tests had clear evidence that the rejection was due to incomplete consent documentation. Although 47 HIV tests were rejected after the change in policy was implemented, none of them was rejected due to lack of consent documentation (Table 1). Table 2 shows the distribution of the characteristics of the patients with HIV tests ordered before and after the modification of the administration procedures for HIV testing.

**Table 1:** Distribution of HIV test results before and after the change in administrative requirements for HIV testing at the San Francisco Department of Public Health medical center, January 2003 to June 2007.

	Before the change in policy	%	After the change in policy	%	Total number of tests	%
**Positive**	336	2.0	109	2.9	445	2.2
**Negative**	12296	72.7	3634	95.9	15930	76.9
**Indeterminate**	13	0.1	1	0	14	0.1
**Confidential** *	3070	18.2	0	0	3070	14.8
**Rejected** **	1204	7.1	47	1.2	1251	6.0
**Total**	16919	100.0	3791	100.0	20710	100.0

* Prior to January 2004, results of HIV testing were reported as “confidential” in the electronic database and no specific results are available.

** 814 (67.6%) HIV tests rejected before the policy change had specific documentation of rejection due to lack consent documentation.

**Table 2:** Distribution of HIV test results by demographic characteristics before and after the change in administrative requirement for HIV testing at the San Francisco Department of Public Health medical center, January 2003 to June 2007

	BEFORE THE CHANGE IN POLICY					AFTER THE CHANGE IN POLICY			
	CONFIDENTIAL (%)	NEGATIVE (%)	POSITIVE (%)	REJECTED (%)	TOTAL	NEGATIVE (%)	POSITIVE (%)	REJECTED (%)	TOTAL
**Sex/gender**									
Male	1242 (19.1)	4385 (67.7)	260 (4.0)	604 (9.3)	6491	1649 (93.8)	83 (4.7)	27 (1.5)	1759
Female	1828 (17.6)	7911 (76.0)	76 (0.7)	600 (5.8)	10415	1985 (97.7)	26 (1.3)	20 (1.0)	2031
**Age**									
18 to 30 years of age	1267 (18.2)	5284 (75.7)	65 (0.9)	362 (5.2)	6982	1288 (97.8)	20 (1.5)	9 (0.7)	1317
31 to 45 years of age	1056 (18.9)	3942 (70.5)	164 (2.9)	428 (7.7)	5594	1096 (94.3)	50 (4.3)	15 (1.3)	1162
>45 years of age	744 (17.2)	3056 (70.7)	107 (2.5)	412 (9.5)	4324	1235 (95.3)	39 (3.0)	22 (1.7)	1296
**Race/ethnicity**									
Asian	292 (16.6)	1328 (75.6)	17 (1.0)	118 (6.7)	1756	426 (99.1)	1 (0.2)	3 (0.7)	430
Black	972 (19.7)	3501 (70.8)	113 (2.3)	352 (7.1)	4943	1056 (95.3)	39 (3.5)	13 (1.2)	1108
Hispanic	778 (15.7)	3835 (77.6)	56 (1.1)	271 (5.5)	4944	1047 (97.0)	24 (2.2)	8 (0.7)	1079
White	764 (18.3)	2877 (69.0)	131 (3.1)	396 (9.5)	4169	868 (93.5)	39 (4.2)	20 (2.2)	928
Other	58 (15.9)	284 (77.6)	2 (0.6)	20 (5.5)	366	105 (99.1)	0 (0.0)	1 (0.9)	106
**Primary language**									
English-speaking	2431 (19.6)	8733 (70.5)	282 (2.3)	933 (7.5)	12389	2580 (95.2)	86 (3.2)	42 (1.6)	2709
Spanish-speaking	464 (14.3)	2588 (79.6)	33 (1.0)	165 (5.1)	3252	694 (97.6)	13 (1.8)	4 (0.6)	711
Other primary language	134 (14.3)	725 (77.1)	11 (1.2)	69 (7.3)	940	279 (98.6)	3 (1.1)	1 (0.4)	283
**Homelessness status**									
Not homeless	2449 (18.7)	9540 (73.0)	238 (1.8)	832 (6.4)	13069	3044 (96.4)	79 (2.5)	35 (1.1)	3159
Homeless	511 (22.4)	1433 (62.8)	68 (3.0)	262 (11.5)	2283	460 (93.1)	23 (4.7)	11 (2.2)	494
**Insurance status**									
Insured	1604 (16.8)	7243 (75.8)	137 (1.4)	571 (6.0)	9561	1992 (96.4)	50 (2.4)	23 (1.1)	2066
Uninsured	1391 (19.7)	4847 (68.7)	192 (2.7)	623 (8.8)	7060	1584 (95.0)	59 (3.5)	24 (1.4)	1667
**Hospital setting**									
Outpatient	2285 (17.7)	9847 (76.2)	212 (1.6)	570 (4.4)	12920	3012 (97.0)	73 (2.4)	18 (0.6)	3104
Inpatient	785 (19.6)	2449 (61.2)	124 (3.1)	634 (15.9)	3999	622 (90.5)	36 (5.2)	29 (4.2)	687

* Prior to January 2004, results of HIV testing were reported as “confidential” in the electronic database and no specific results are available.

We found that male sex, age over 45 years, and lack of insurance were associated with a higher odds of having an HIV positive test result and a higher odds of having an HIV test rejected due to lack of consent documentation in the period prior to the consent policy change (Table 3). Other factors associated with a higher odds of having an HIV positive test result were age between 30 and 45 year-old, white race/ethnicity, and homelessness status (Table 3). Speaking a language other than English or Spanish was also associated with an increased odds of having an HIV test rejected due to lack of consent documentation in the period before the change in policy(Table 3).

**Table 3:** Factors associated with rejected tests due to lack of written documentation of HIV consent and factors associated with HIV positive test results, San Francisco Department of Public Health medical center, January 2004 to April 2006.

	HIV tests rejected due to lack of consent documentation			HIV positive test result		
	Adjusted* OR	95% CI	P	Adjusted* OR	95% CI	P
**Female**	1.00			1.00		
**Male**	**1.34**	**1.14–1.57**	**<0.001**	**5.76**	**4.49–7.40**	**<.001**
**Age18–30 year-old**	1.00			1.00		
**Age 31–45 year-old**	NS	NS	NS	**2.45**	**1.87–3.20**	**<.001**
**Age>45 year-old**	**1.24**	**1.06–1.45**	**<0.01**	**1.40**	**1.04–1.89**	**0.027**
**White**	1.00			1.00		
**Hispanic**	NS	NS	NS	**0.47**	**0.35–0.62**	**<. 001**
**Other ethnicity**	NS	NS	NS	**0.29**	**0.09–0.93**	**0.037**
**English**	1.00			1.00		
**Other languages**	**1.58**	**1.15–2.17**	**<0.01**	**0.36**	**0.17–0.77**	**0.008**
**Insured**	1.00			1.00		
**Uninsured**	**1.24**	**1.07–1.45**	**0.005**	**1.30**	**1.06–1.58**	**0.01**
**Not homeless**	1.00			1.00		
**Homeless**	**NS**	NS	NS	**1.40**	**1.13–1.74**	**.002**
**Outpatient**	1.00			**1.00**		
**Inpatient**	**4.45**	**3.80–5.21**	**<0.001**	**0.44**	**0.35–0.55**	**<0.001**

NS = not significant,

* Adjusted for gender, age category, insurance status, homelessness status, race/ethnicity, and testing venue.

**Figure 1:**
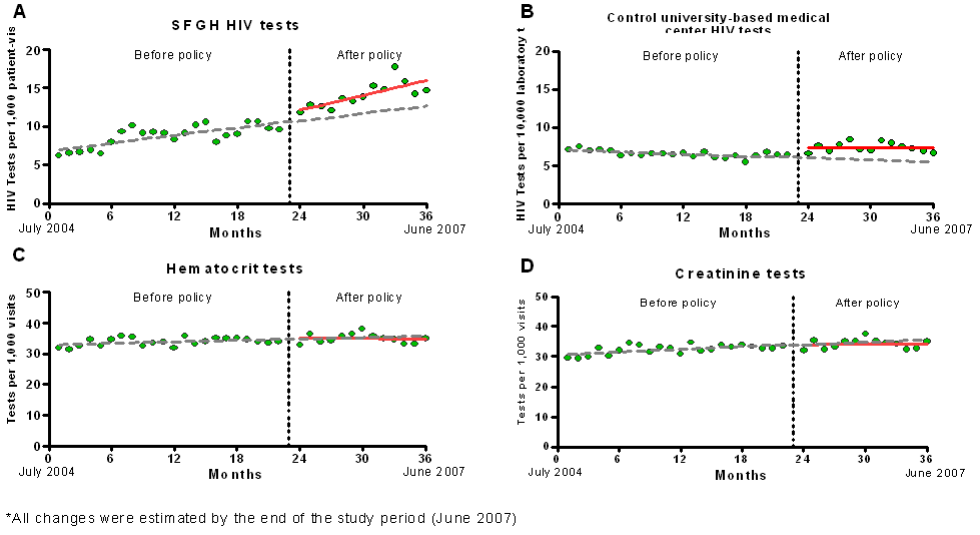


Our time-series analysis documented an increasing trend in the monthly rates of HIV tests per 1000 patient-visits before the policy change (average monthly increase of 0.19 [CI, 0.01–0.38], p = 0.04) (Figure 1A). This analysis was adjusted for age, race, language, gender, homelessness status, insurance and health care setting. By June 2007, one year after the policy change, the average monthly rate of HIV tests per 1000 patient-visits had increased 4.38 (CI, 2.17–6.60, p<0.001) over the number predicted if the change had not occurred (Figure 1A). The monthly average of new positive HIV tests increased from 8.9 (CI, 6.3–11.5) to 14.9 (CI, 10.6–19.2, p<0.001). An increasing trend in HIV testing was not found in the comparison medical center where a testing policy change had not occurred (average increase of 0.09 tests per month per 10000 laboratory tests performed [CI, −0.10–0.27], p = 0.35) (Figure 1B). Moreover, no increases in monthly rates of laboratory testing for tests other than HIV were found within our institution (Figures 1C and 1D).

**Figure 2:**
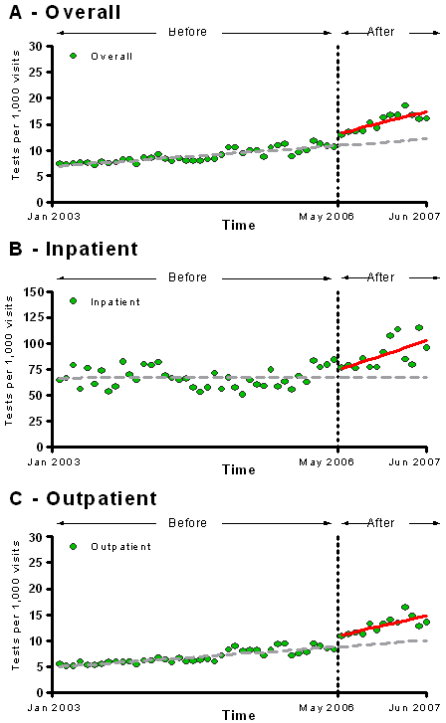


A substantial increase in the proportion of HIV testing performed in the outpatient setting followed the implementation of the new policy (from 76.3% to 81.8%, p<0.05). However, increases in monthly HIV testing were seen both in the outpatient and inpatient settings. By the end of the study period, there were 3.26 (CI, 1.45–5.07, p<0.001) and 29.80 (12.01–47.60, p<0.001) monthly HIV tests per 1000 patient-visits more than expected in the outpatient and inpatient settings, respectively (Figures 2A and 2B). Although the monthly HIV testing rates per 1000 patients were higher in the inpatient setting during the entire study period, the increasing trends observed after the policy change in the outpatient and inpatient settings were not significantly different when compared to each other (p = 0.10).

**Figure 3:**
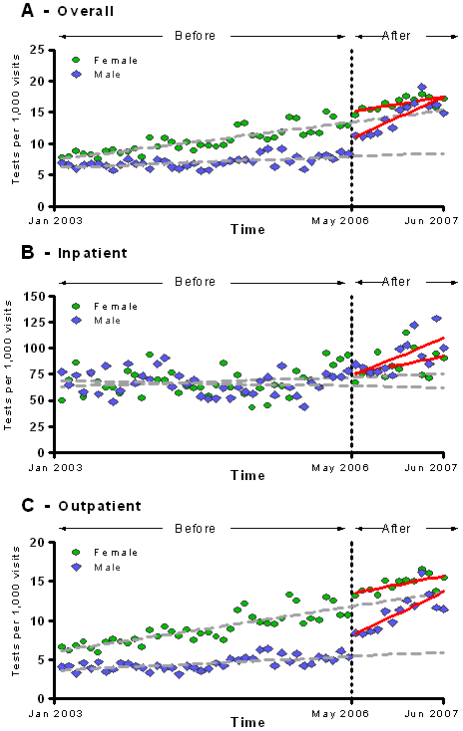


Before the policy change was implemented, women had a significantly increasing trend in the monthly rates of HIV testing (monthly average increase of 0.11 [CI, 0.06–0.17] tests per 1000 patient-visits, p<0.001) (Figure 3). However, after the policy was implemented, the HIV testing trend was 2.7 times higher in men (0.32 tests per month per 1000 patient-visits [CI 0.30–0.93]) than women (0.12 tests per month per 1000 patient-visits [CI, −.078–0.32], p<0.01) (Table 4).

**Table 4:** Mean change in the number of HIV tests per month per 1000 patient visits by the end of the study period, San Francisco Department of Public Health medical center, June 2006 to June 2007.

	Overall		Inpatient setting		Outpatient setting	
	Mean HIV tests per month per 1000 patient visits over the expected number of tests 13 months after the change in policy (95% confidence interval)	P value	Mean HIV tests per month per 1000 patient visits over the expected number of tests 13 months after the change in policy (95% confidence interval)	P value	Mean HIV tests per month per 1000 patient visits over the expected number of tests 13 months after the change in policy (95% confidence interval)	P value
Male	6.94 (3.57–10.31)	<0.001	47.75 (25.59–69.92)	<0.001	5.98 (3.02–8.94)	<0.001
Female	1.50 (−0.03–3.03)	0.055	15.70 (−3.56–34.97)	0.108	1.40 (−0.13–2.93)	0.072
18 to 30 years of age	2.02 (−1.77–5.80)	0.289	15.97 (−14.14–46.08)	0.291	2.40 (−1.30–6.11)	0.199
31 to 45 years of age	7.69 (4.85–10.53)	<0.001	28.75 (4.68–52.82)	0.020	6.54 (3.78–9.30)	<0.001
>45 years of age	3.37 (1.37–5.38)	0.001	38.36 (17.94–58.77)	<0.001	3.61 (1.89–5.33)	<0.001
Asian	2.80 (1.37–4.23)	<0.001	−0.29 (−22.64–22.05)	0.979	3.02 (1.69–4.35)	<0.001
Black	5.58 (2.11–9.04)	0.002	37.21 (8.61–65.80)	0.012	6.19 (2.98–9.40)	<0.001
Hispanic	1.56 (−0.49–3.61)	0.132	46.01 (21.72–70.29)	<0.001	0.74 (−1.12–2.61)	0.427
White	5.58 (2.95–8.21)	<0.001	34.50 (6.68–62.32)	0.016	4.90 (2.41–7.39)	<0.001
English-speaking	5.04 (2.40–7.69)	<0.001	26.17 (5.56–46.78)	0.014	5.91 (3.47–8.36)	<0.001
Spanish-speaking	−0.95 (−3.31–1.40)	0.419	40.03 (12.78–67.28)	0.005	−1.48 (−3.78–0.81)	0.200
Other primary language	2.69 (1.16–4.22)	0.001	16.79 (−9.85–43.42)	0.211	2.15 (0.70–3.60)	0.004
Not homeless	−0.59 (−2.64–1.45)	0.563	29.90 (13.03–46.78)	0.001	0.17 (−0.52–1.85)	0.844
Homeless	2.29 (−1.51–6.09)	0.232	46.66 (13.50–79.82)	0.007	5.74 (2.61–8.88)	0.001
Insured	1.77 (0.15–3.38)	0.032	33.01 (16.04–49.98)	<0.001	1.36 (−0.22–2.95)	0.089
Uninsured	6.53 (3.78–9.28)	<0.001	33.64 (7.68–59.61)	0.012	5.88 (3.34–8.42)	<0.001

**Figure 4:**
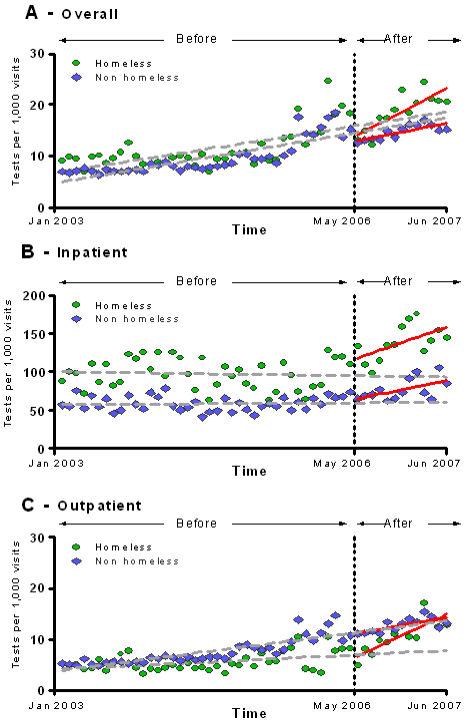


After the change in policy, homeless individuals had significantly increasing monthly HIV testing rates in the inpatient and outpatient settings (Figure 4) (Table 4). However, the effect of the policy change had a much stronger effect on HIV testing rates among the homeless in the outpatient setting when compared to non-homeless in the outpatient setting. By the end of the study period, homeless individuals tested for HIV in the outpatient setting had 5.74 (CI, 2.61–8.88, p = 0.001) HIV tests per 1000 patient-visits more than their expected rates. Contrary to this finding, non-homeless individuals in the outpatient setting did not experience a significant increase in monthly HIV testing rates per 1000 patient-visits more than their expected rates (0.17 [CI, −0.52–1.85], p = 0.20) (Figure 4C).

**Figure 5:**
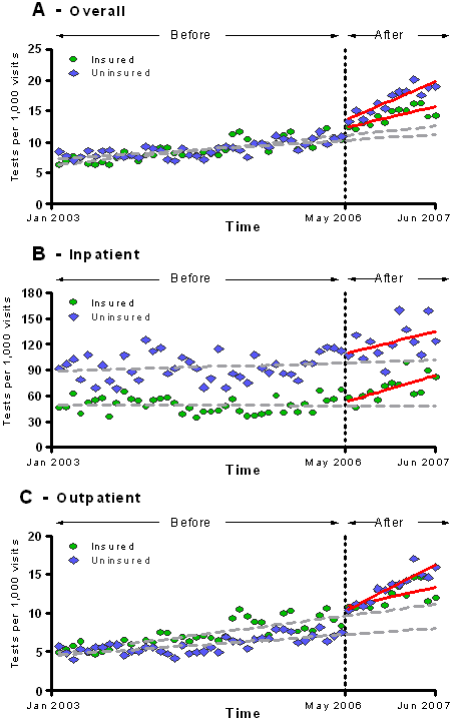


The monthly average increase in HIV testing rates at the end of the study period was significantly higher among uninsured individuals (6.53 tests per 1000 patient-visits [CI, 3.78–9.28]) than among insured individuals (1.77 tests per 1000 patient-visits [CI, 0.15–3.38]) (Figure 5) (Table 4). In the outpatient setting, the increasing trend of HIV testing among the uninsured was significantly higher than among the insured (0.28 [CI, 0.03–0.54], p = 0.03) (Figure 5C).

**Figure 6:**
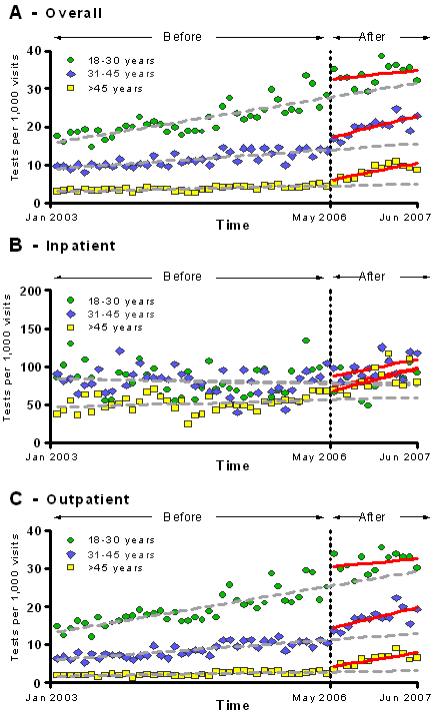


Age-stratified analyses revealed consistent increases in HIV testing rates across age groups and in both the outpatient and inpatient settings after the policy change (Table 4). The increasing trends were similar across the various age categories (Figure 6).

**Figure 7:**
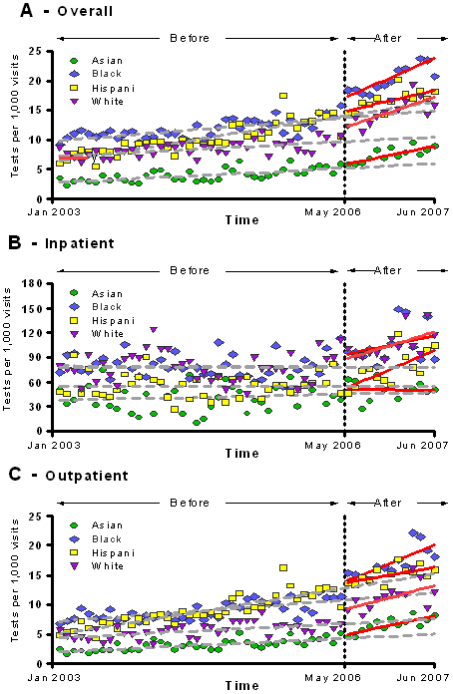


When the analysis was stratified by race/ethnicity, an increased number of HIV tests per month per 1000 patient visits after the change in policy was found among Whites, African Americans and Asians, but not among Hispanics (Table 4). However, increasing HIV testing trends across racial/ethnic groups were similar when compared to each other (Figure 7A). A significant increase in HIV testing rates over the expected values among Hispanics was seen in the inpatient setting (3.97 tests per month per 1,000 visits [CI, 1.47–6.48], p<0.01) (Figure 7B). Asians had an increasing trend HIV testing rates over the expected trend in the outpatient setting (0.20 tests per month per 1,000 visits [CI, 0.07–0.33], p<0.01) after the policy change (Figure 7C).

**Figure 8:**
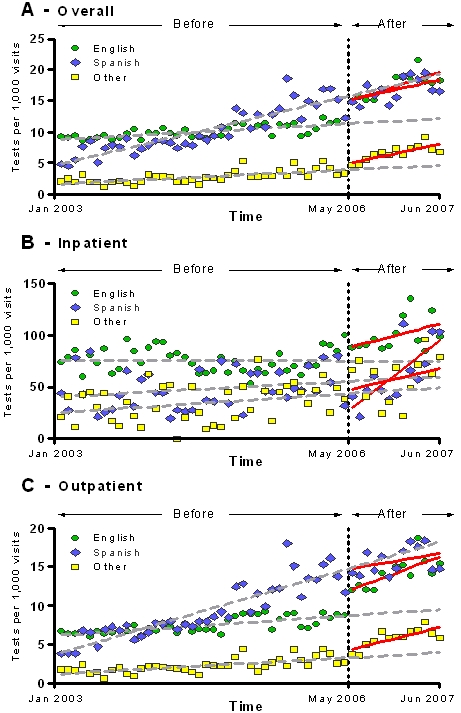


After the policy change, increasing HIV testing rates were seen regardless of their primary language (Figure 8). However, by the end of the study period, only patients speaking English and patients speaking a primary language other than English or Spanish had a significant increase in the average number of HIV tests per month per 1000 visits over the expected number of tests (5.04 [2.40–7.69] and 2.69 [1.16–4.22] respectively) (Table 4).

## Discussion

Our analyses demonstrate a sustained increase in monthly HIV testing rates one year after an administrative policy change in requirements for HIV testing. Elimination of the separate laboratory requisition form and documentation of patient written consent for HIV testing was associated with increased monthly HIV testing rates. These observations remained consistent after adjustment for sex, age, race/ethnicity, hospital setting, homelessness status and insurance status. Given that no changes in monthly HIV testing rates were found in a comparison medical center in San Francisco during the same period, the increased rates reported at our institution are not likely to be related to changes in HIV testing practices in the community or increased awareness of HIV screening recommendations at the patient level. Similarly, given that no increases were found in monthly testing rates for tests other than HIV, it is unlikely that changes in general testing practices within our institution could have accounted for the increases. More importantly, increased testing, particularly among underserved populations at high risk for HIV infection, led to a significant increase in positive HIV tests after the policy change.

One year after the policy change, we continued to observe sustained increases in overall monthly HIV testing rates among all the subpopulations included in this study. Although we believe this increase, for the most part, is still attributable to the elimination of the separate laboratory test requisition form and of the requirement for a patient signature to document consent, there were two other important events during the study period that might have contributed to this effect. First, as part of efforts to increase HIV testing, same-day HIV testing was implemented at the SFDPH medical center in February 2007. Same-day HIV testing has allowed the implementation of HIV testing and screening programs in settings with brief patient encounters, where HIV testing was not previously offered (e.g. emergency department and urgent care clinic) [11]. Although we acknowledge that those events could have contributed to the sustained increases in HIV testing rates, sensitivity analysis excluding HIV tests performed at the emergency department and urgent care clinic suggests that such interventions did not alter the increasing trend in HIV testing rates established before that point (data not shown). Secondly, the publication of our preliminary findings in a major medical journal on March 2007 [4] and the subsequent media attention might have increased awareness of recommended HIV screening and testing practices, both in the general population and among physicians, leading to an increase in self referral or referral by physicians outside the SFDPH medical system for HIV testing. We did not find a difference in the HIV testing trends before and after the publication of our preliminary findings (data not shown). Although the limited number of data points after this event prevents us from drawing strong conclusions, the lack of increasing HIV testing rates at the comparison medical center suggests that the impact of that event was limited.

As reported by others, we found that most HIV testing at our institution was performed in the outpatient setting. The substantial increase in the proportion of HIV testing performed in the outpatient setting followed the implementation of the new policy suggests that the increase in monthly HIV testing rates occurred mostly due to HIV testing incorporated into routine medical care. Although increasing HIV testing rates across all populations might have led to increased HIV testing among low risk populations, screening of populations without traditional risk factors for HIV infection is supported by the recent CDC recommendations for universal HIV testing in health care settings [2], [3].

Given that many rejected HIV tests occurred in the inpatient setting and were followed by a valid HIV test before discharge, we only included HIV tests that were not followed by a valid HIV test within the next 4 weeks after initial testing. This algorithm allowed us to include and analyze rejected HIV tests that resulted in missed opportunities for diagnosis. We found that before the change in testing policy was implemented, populations at higher risk for HIV infection were facing increased structural barriers to testing [7], [8]. Male sex and lack of insurance were factors significantly associated with both a higher odds of HIV infection and a higher odds of having an HIV test rejected due to the lack of consent documentation. Speaking a language other than English or Spanish was also significantly associated with increased odds of having an HIV test rejected due to the lack of consent documentation, and White race, and homelessness status were associated with a higher odds of HIV infection. By decreasing barriers to HIV testing, populations with the highest likelihood of HIV test rejection due to lack of consent documentation and HIV positive test results–particularly men, homeless persons and uninsured patients in our sample–had the greatest increase in monthly HIV testing rates.

Before June 2005, SFDPH medical center had a State of California funded HIV testing service that performed HIV counseling and obtained consent hospital-wide, including in the prenatal clinic. The availability of that service could have contributed to the overall slight increase in HIV testing observed between January 2003 and June 2005, after which this program was discontinued. However, monthly HIV testing rates continued to increase after June 2005, primarily among women. After this program ended, prenatal nurses were trained to offer HIV testing to and obtain consent from all pregnant patients while conducting initial prenatal intake sessions. These efforts led to routine HIV testing of nearly all women in prenatal care by May 2006, when the new consent policy was implemented. The fact that testing of this population was essentially maximized before the implementation of the new policy may explain the larger increases in HIV testing observed in men, compared to women, after the policy change. However, because the monthly HIV testing rates in women continued to increase after the policy change, despite having maximized the testing of women in obstetrical care, may suggest that most of the additional HIV testing observed in women after the policy change occurred as part of routine medical care.

We observed similar sustained increases in monthly HIV testing rates in all racial/ethnic groups and regardless of insurance status. Racial/ethnic minorities and the uninsured are populations at high risk for HIV infection who traditionally have been difficult to reach [9], [10]. Our findings suggest that after decreasing the barriers to HIV testing, these underserved groups experienced the highest increases in HIV testing rates and HIV case detection.

Differences in the data management and analysis used in this study led to slightly different results than reported previously by our group [4]. First, in this report we calculated the monthly rates of HIV testing using only patient-visits to health care settings in which HIV screening is routinely performed. Similarly, we excluded all tests ordered at the HIV primary care clinic because we believe that those tests do not reflect HIV screening practices, as all new patients seen at this clinic are re-tested to confirm their HIV infection status. The inclusion of the monthly HIV testing rates during 2003 in this report also increased the HIV trend in monthly HIV testing rates seen before the policy change compared to our previous report. Similarly, the use of a different algorithm to define new HIV cases and more extensive retrospective data collection in search of any evidence of previous diagnosis of HIV infection among the cases testing positive may have changed the mean number of new HIV cases detected per month.

Certain limitations to our study should be acknowledged. The observational nature of our study prevents us from concluding that there is a cause-effect relationship between the change in administrative policy and the increase in HIV testing and case finding seen afterwards. Given that our definition of “new cases” of HIV infection was limited by the data available in the SFDPH medical center, it is impossible for us to determine if those cases were truly new cases or if they had been previously found to be HIV antibody positive outside our system. Unfortunately, given the lack of reliable data regarding certain HIV risk factors in our database, we were not able to analyze HIV testing trends and HIV case detection among other populations at high risk for HIV infection (e.g. intravenous drug users, men who have sex with men, patients with history of prior sexually transmitted infections, etc.). However, our results (particularly the results from the analysis of the overall HIV testing trend after the change in policy) were consistent with the CDC guidelines for HIV testing in health care settings which recommend universal screening over risk-based testing [2]. Similarly, the inability to accurately calculate the HIV testing rates at the control hospital limits the extent to which we can interpret the comparison with the HIV testing trends at our own institution. As previously discussed, other factors (the new availability of rapid HIV tests, the publication the results of a previous study, patient self referral, etc) could also have contributed to the increases in HIV testing rates reported here. However, the large number of events assessed in this study and the 13-month follow up, allowed us to perform a comprehensive analysis of the effect of the policy change on monthly HIV testing rates showing a strong and consistent effect in various subgroups. Similar findings using different statistical approaches and the use of internal and external controls increase our confidence in our results.

### Conclusion

An administrative policy change that eliminated a separate laboratory test requisition form and a patient-signed consent document was associated with a sustained increase in HIV testing and an increase in HIV case detection one year later. Although increases in HIV testing were seen across all the populations studied, certain subgroups at high-risk for HIV infection had the greatest increases. Although further studies in other populations and using different designs are required to confirm these findings, our study supports the benefits of current efforts to reduce administrative barriers to HIV testing as means to increase HIV case-detection.
